# Twist drill craniostomy for traumatic acute subdural hematoma in the elderly: case series and literature review

**DOI:** 10.1186/s41016-019-0157-8

**Published:** 2019-05-07

**Authors:** Pei-kun Huang, Yong-zhong Sun, Xue-ling Xie, De-zhi Kang, Shu-fa Zheng, Pei-sen Yao

**Affiliations:** 1 Department of Neurosurgery, The First Affiliated Hospital of Fujian Medical University, NO. 20 Chazhong Road, Taijiang District, Fuzhou, 350004 China; 2 Fujian Medical University, Fuzhou, 350004 China; 3 Department of Neurosurgery, Hui’an County Hospital, Hui’an, 362100 China; 4 The First Clinical Medical College of Fujian Medical University, NO. 20 Chazhong Road, Taijiang District, Fuzhou, 350004 China

**Keywords:** Traumatic acute subdural hematoma, Cerebral herniation, Twist drill craniostomy, Pre-injury antiplatelet therapy, The elderly

## Abstract

**Background:**

A large craniotomy is usually the first choice for removal of traumatic acute subdural hematoma (TASDH). To date, few studies have reported that TASDH could be successfully treated by twist drill craniostomy (TDC) alone or combined with instillation of urokinase. We aimed to define the TDC for the elderly with TASDH and performed literature review.

**Case presentation:**

A total of 7 TASDH patients, who were presented and treated by TDC in this retrospective study between January 2009 and May 2017, consisted of 5 men and 2 women, ranging in age from 65 to 89 (average, 78.9) years. The patients’ baseline characteristics, including age, sex, medical history, received ventriculoperitoneal shunt for hydrocephalus or not, reason for avoiding or refusing large craniotomy, preoperative Glasgow Coma Scale (GCS), suffered from cerebral herniation or not, the location of TASDH, imaging characteristics of TASDH in CT scan, injury/surgery time interval, midline shift, preoperative neurologic deficit, operation time, and infusions of urokinase or not, were collected. The postoperative GCS, postoperative neurologic deficit, rebleeding or not, intracranial infection, and modified Rankin Scale (mRS) at 6 months after surgery were analyzed to access the safety and efficacy of evacuation with TDC. The results showed that the mean time interval from injury to TDC was 68.6 min (30–120 min). The mean distance of midline shift was 14.6 mm (10–20 mm). The preoperative GCS in all patients ranged from 4 to 13(median, 9). The mean duration of the operation was 14.4 min (6–19 min). Postoperative CT scan showed that hematoma evacuation rate was more than 70% in all cases. There were no cases of acute rebleeding and intracranial infection after TDC. No cases presented with chronic SDH at the ipsilateral side within 6 months after being treated by TDC alone or combined with instillation of urokinase. Favorable outcomes were shown in all cases (mRS scores 0–2) at 6 months after surgery.

**Conclusions:**

TASDH in the elderly could be safely and effectively treated by TDC alone or combined with instillation of urokinase, which was a possible alternative for the elderly.

## Background

Traumatic acute subdural hematoma (TASDH) caused by fall in the elderly patients is growing with the aging populations. A large craniotomy is usually the first choice for removal of TASDH. However, it is always considered to be unsuitable, owing to the increasing age, the pre-injury antiplatelet therapy (APT), and/or comorbid burden. Many neurosurgeons are hesitant in offering aggressive management for these patients, which poses a therapeutic dilemma. The security and effectiveness of twist drill craniostomy (TDC) have been confirmed in the treatment of chronic subdural hematoma [1–3]. However, its security and effectiveness have not been verified in TASDH. TDC was usually chosen for saving valuable time in the patients with TASDH and cerebral herniation before evacuation of subdural hematoma via decompressive craniectomy. To date, however, few studies have reported that TASDH could be successfully treated by TDC alone or combined with instillation of urokinase. In this case series, the clinical data of the elderly patients, who underwent urgent TDC alone or combined with infusions of urokinase for the removal of TASDH, were collected and analyzed retrospectively. And we also performed literature review.

## Case presentation

This study was performed in the First Affiliated Hospital of Fujian Medical University and Hui’an County Hospital. All procedures performed in this study involving human participants were in accordance with the 1964 Helsinki declaration and approved by the ethics committee of the First Affiliated Hospital of Fujian Medical University and Hui’an County Hospital. All patients provided written informed consent.

A total of seven TASDH patients treated by TDC were enrolled in this retrospective study between January 2009 and May 2017. In our institution, the preferred scheme choice for the removal of TASDH is a large craniotomy. The following factors contribute to avoiding and/or refusing large craniotomy or decompressive craniectomy for TASDH; however, TDC was proposed when the following inclusion criteria were met: (1) old age (≥ 65 years), (2) cerebral atrophy in imaging appearance, (3) TASDH without severe brain swelling or obvious brain contusion and laceration, and (4) the pre-injury antiplatelet therapy(APT) and/or comorbid burden including cognitive dysfunction, cerebral infarction(CI), congestive heart failure(CHF), and cardiopulmonary complications. The exclusion criteria were severe brain swelling and/or obvious brain contusion and laceration.

The patients’ baseline characteristics, including age, sex, medical history, reasons for TASDH, reason for avoiding or refusing large craniotomy, hematoma volume, preoperative Glasgow Coma Scale (GCS), suffered from cerebral herniation or not, the location of TASDH, imaging characteristics of TASDH in CT scan, injury/surgery time interval, midline shift, preoperative neurologic deficit, operation time, infusions of urokinase or not, and total drainage time, were collected. Postoperative neurologic deficit, rebleeding or not, intracranial infection, the GOS at 6 months after surgery, and mRS at 6 months after surgery were analyzed to access the safety and efficacy of evacuation with TDC.

**Fig. 1:**
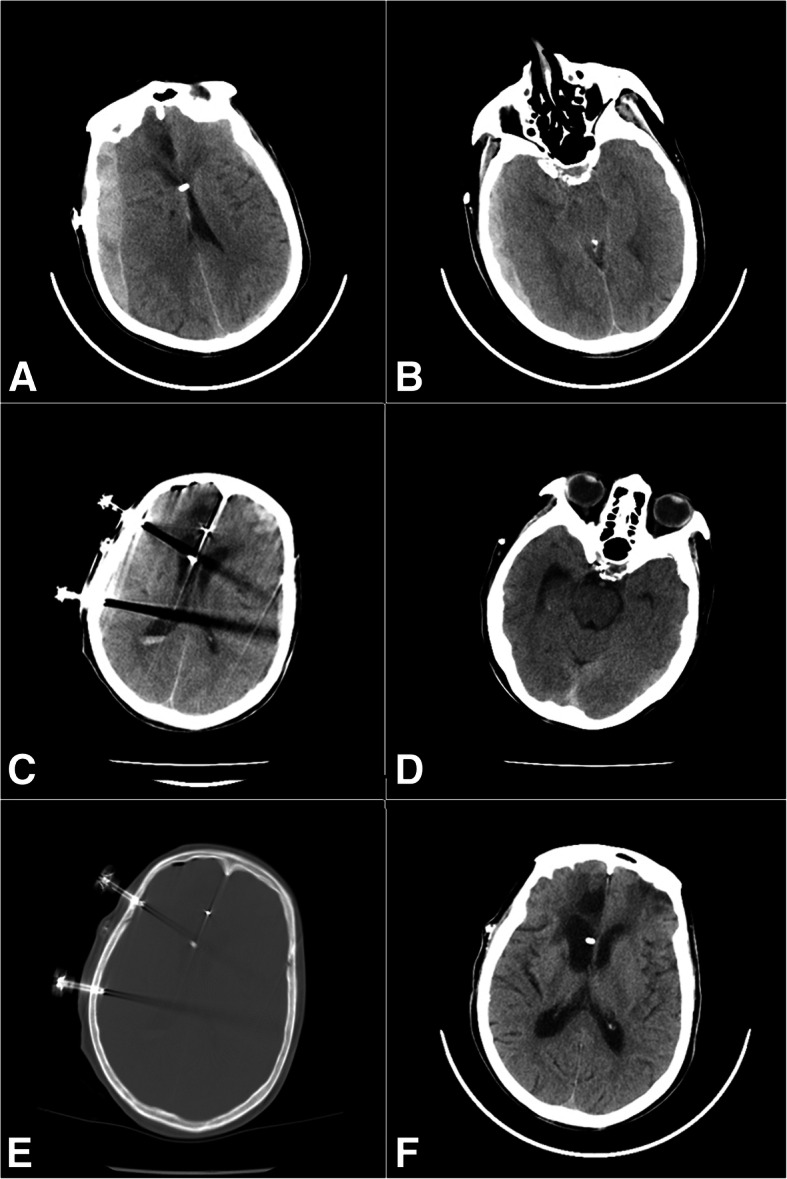
Preoperative brain CT scan showed a large traumatic acute subdural hematomas (TASDH) in the right frontal-temporal-parietal region (**a**, **b**). Brain CT scan revealed there was a small amount of hematoma in the right subdural space and expansion (**c**, **d**). 1 day after surgery. CT showed the puncture needle passing through the skull and dura and just reaching the edge of the hematoma cavity (**e**). A follow-up brain CT scan 6 months later after TDC (**f**)

**Fig. 2:**
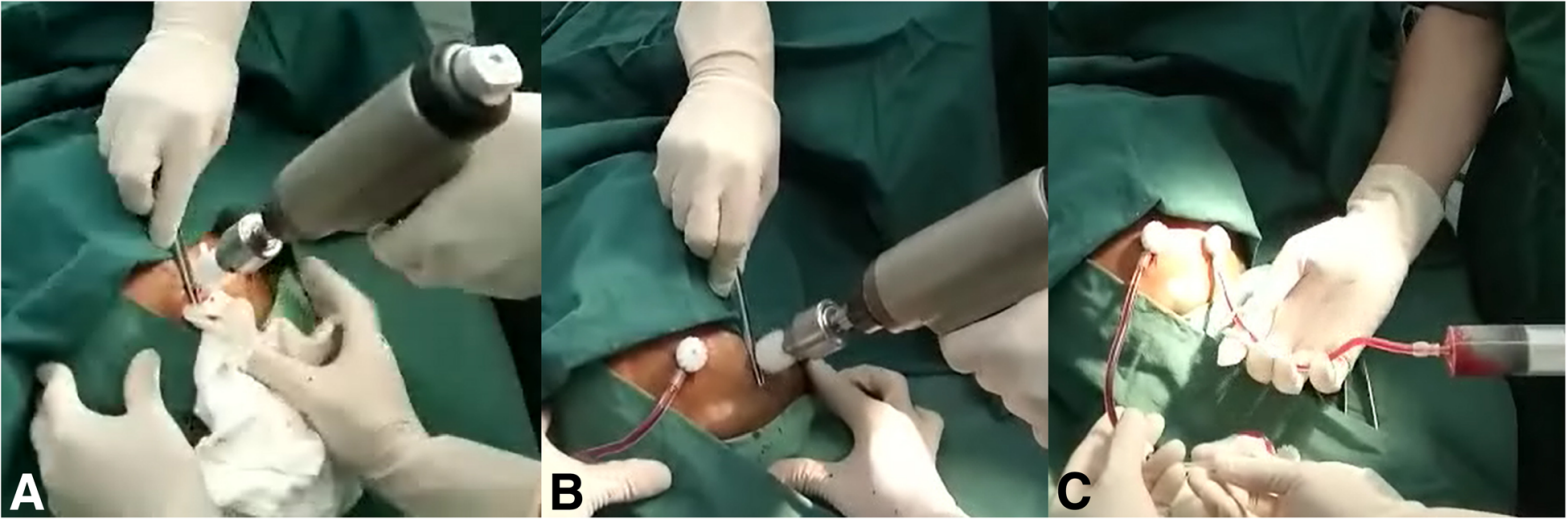
The two target points were established in the area according to the brain CT scan, which were located in the maximum level of the hematoma, and chosen to prevent injury to superficial temporal artery and its branches and middle meningeal artery (**a**-**b**). After the inner needle was retrieved, bloody fluid, which was not coagulable, could be extracted from the subdural regions respectively (**c**)

The operative procedure of TDC was urgently performed. Patients were in need of a supine position, the head was turned to the opposite side of the subdural hematoma, and the two target points were established in the area according to the brain CT scan, which were located in the maximum level of the hematoma, and chosen to prevent injury to superficial temporal artery and its branches and middle meningeal artery (Fig. 2a–b). After the local infiltration anesthesia with 2% lidocaine, a YL-1 puncture needle (WanTeFu Medical Apparatus Co., Ltd., Beijing, China) of suitable length was used and fixed to the electric drill [4], which facilitated the drill passing through the skull and dura, and just reaching the edge of the hematoma cavity(Fig. 1e). The intracranial depth of the needle was limited to 7 mm. After the inner needle was retrieved, blood clots mixed with cerebrospinal fluid (the cases with damaged arachnoid membrane caused by TBI) or bloody fluid, which was not coagulable, could be extracted from the subdural regions respectively (Fig. 2c), then the steel tubes were connected to a closed drainage system. If the drainage tube was not blocked, liquid pulsation would appear in the tube. Then, we could raise the tube to a vertical position, which would facilitate knowing the intracranial pressure preliminarily. If a volume greater than 20 ml of blood clots mixed with cerebrospinal fluid or bloody fluid was extracted from the drainage tube during operation, the patients did not received installation of urokinase. Whether the installation of 5000 to 10,000 IU of urokinase (Tianjin Biochemical Pharmaceutical Co., Ltd) in 5 ml of saline were postoperatively performed from the drainage tube according the CT imaging situation, if a volume less than 20 ml were extracted intraoperatively, the patients received installation of 5000–10,000 IU of urokinase in 2 ml of saline and the clamping tube should be opened 2–4 h later. It could be repeated every 6–8 h at bedside [5]. Coagulation monitoring was carried out during the period of installation.

The hematoma volume = *π*/6 × long diameter (mm) × wide diameter (mm) × layer thickness (mm) × layer number. Hematoma evacuation rate = hematoma volume after TDC/hematoma volume before TDC × 100%.

The clinical characteristics and outcome of the TASDH patients treated by TDC were summarized in Table 1. The reasons for avoiding and/or refusing large craniotomy, large craniotomy, or decompressive craniectomy were old age, the pre-injury antiplatelet therapy(APT), retaining the VP shunt tube, and comorbid burden including hypertension, diabetes mellitus (DM), cognitive dysfunction, CI, CHF, and cardiopulmonary complications. The patients population consisted of 5 men and 2 women, ranging in age from 65 to 89(average, 78.9) years. Three patients have undergone ventriculoperitoneal shunt procedure because of subarachnoid hemorrhage, spontaneous intracranial hemorrhage, or traumatic hydrocephalus before. There were two patients with cerebral herniation before TDC. Preoperative CT scans showed mixed high-low densities in four patients and high densities in three. The mean time interval from injury to surgery was 68.6 min (30–120 min). The mean distance of midline shift was 14.6 mm (10–20 mm). TASDH in the four patients with mixed high-low densities (shown in preoperative CT scans) was easily extracted, while that in the three patients with high densities was not. The preoperative GCS in all patients ranged from 4 to 13 (median, 9). The mean duration of the operation was 14.4 min (6–19 min). Postoperative CT scan showed that hematoma evacuation rate was more than 70% in all cases. There were no cases of acute rebleeding and intracanial infection after TDC. No cases presented with chronic SDH at the ipsilateral side within 6 months after being treated by TDC alone or combined with the instillation of urokinase. Favorable outcomes were shown in all cases (mRS scores 0–2) at 6 months after surgery. Table 2 demonstrates that TDC for TASDH is safe, controllable, and effective.

**Table 1:** Clinical characteristics and outcome of the patients treated with twist drill craniostomy for traumatic acute subdural hematoma

Case	Age	Sex	Medical history	Reason for avoiding or refusing large craniotomy	Reasons for TBI	The location of TASDH	Hematoma volume (ml)	Imaging characteristics of TASDH	Preoperative GCS	Cerebral herniation	Injury/surgery time interval (min)	Midline shift (mm)	Postoperative deficit	Operation time (min)	Infusions of urokinase	Total drainage time (*d*)	Hematoma evacuation rate (%)	Rebleeding	Intrcanial infection	GOS at 6 months after surgery	mRS at 6 months after surgery
1	71	F	aSAH, CHF, HT, DM	CHF, old age, pre-injury antiplatelet therapy,	Stumble	Right	81	Mixed high-low densities	4	+	60	10	Full recovery	13	−	1	85	−	−	5	0
2	83	M	CI, DM	CI, pre-injury antiplatelet therapy, old age	Stumble	Left	54	Mixed high-low densities	8	−	50	12	Full recovery	18	+	5	70	−	−	5	0
3	85	F	HT, DM, sICH	DM, medical expenses, old age	Stumble	Left	73	High densities	4	+	70	20	Partial recovery	19	+	2	74	−	−	5	1
4	87	M	CI, CHF	CI, CHF, old age, pre-injury antiplatelet therapy	Stumble	Left	67	High densities	12	−	30	15	Full recovery	16	+	4	81	−	−	5	0
5	72	M	CI, HT, CHF, DM	CI, CHF, DM, pre-injury antiplatelet therapy	Stumble	Right	53	Mixed high-low densities	10	−	60	18	Full recovery	14	−	2	87	−	−	5	0
6	89	M	HT, TBI, CHF	CHF, old age	Stumble	Left	62	High densities	9	−	90	12	Partial recovery	6	−	1	79	−	−	4	2
7	65	M	HT, CHF, DM	CHF, old age	Stumble	Left	78	Mixed high-low densities	13	−	120	15	Full recovery	15	−	1	90	−	−	5	0

*CI* cerebral infarction, *CHF* congestive heart failure, *DM* diabetes mellitus, *GCS* Glasgow Coma Scale, *GOS* Glasgow Outcome Scale, *HT* hypertension, *TASDH* traumatic acute subdural hematoma, *TBI* traumatic brain injury, *aSAH* aneurismal subarachnoid hemorrhage, *sICH* spontaneous intracranial hemorrhage

**Table 2:** The basic condition of the seven cases before and after operation

	Before operation	After operation							
		1 day	2 days	3 days	4 days	5 days	6 days	7 days	14 days
Case 1									
Body temperature	Normal	Normal	Normal	Normal	Normal	Normal	Normal	Normal	Normal
GCS	4	9	13	15	15	15	15	15	15
Pupil diameter (left, right) (mm)	3, 4	2.5, 2.5	2.5, 2.5	2.5, 2.5	2.5, 2.5	2.5, 2.5	2.5, 2.5	2.5, 2.5	2.5, 2.5
Pupilary light reflex	–	+	++	++	++	++	++	++	++
Postoperative drainage volume (ml)	/	45	/	/	/	/	/	/	/
Case 2									
Body temperature	Normal	Normal	Normal	Normal	Normal	Normal	Normal	Normal	Normal
GCS	8	8	8	9	10	11	12	14	15
Pupil diameter (left, right) (mm)	3, 3	3, 3	3, 3	3, 3	3, 3	3, 3	3, 3	3, 3	3, 3
Pupilary light reflex	++	++	++	++	++	++	++	++	++
Postoperative drainage volume (ml)	/	140	305	120	110	117	/	/	/
Case 3									
Body temperature	Normal	Normal	Normal	Normal	Normal	Normal	Normal	Normal	Normal
GCS	4	7	9	9	10	11	13	14	15
Pupil diameter (left, right) (mm)	4, 3	3, 3	3, 3	3, 3	3, 3	3, 3	3, 3	3, 3	3, 3
Pupilary light reflex	+	+	++	++	++	++	++	++	++
Postoperative drainage volume (ml)	/	110	30	/	/	/	/	/	/
Case 4									
Body temperature	Normal	Normal	Normal	Normal	Normal	Normal	Normal	Normal	Normal
GCS	12	15	15	15	15	15	15	15	15
Pupil diameter (left, right) (mm)	3, 3	3, 3	3, 3	3, 3	3, 3	3, 3	3, 3	3, 3	3, 3
Pupilary light reflex	++	++	++	++	++	++	++	++	++
Postoperative drainage volume (ml)	/	390	330	/	/	/	/	/	/
Case 5									
Body temperature	Normal	Normal	Normal	Normal	Normal	Normal	Normal	Normal	Normal
GCS	10	15	15	15	15	15	15	15	15
Pupil diameter (left, right) (mm)		2.5, 2.5	2.5, 2.5	2.5, 2.5	2.5, 2.5	2.5, 2.5	2.5, 2.5	2.5, 2.5	2.5, 2.5
Pupilary light reflex		++	++	++	++	++	++	++	++
Postoperative drainage volume (ml)	/	310	/	/	/	/	/	/	/
Case 6									
Body temperature	Normal	Normal	Normal	Normal	Normal	Normal	Normal	Normal	Normal
GCS	9	15	15	15	15	15	15	15	15
Pupil diameter (left, right) (mm)	2.5, 2.5	2.5, 2.5	2.5, 2.5	2.5, 2.5	2.5, 2.5	2.5, 2.5	2.5, 2.5	2.5, 2.5	2.5, 2.5
Pupilary light reflex	++	++	++	++	++	++	++	++	++
Postoperative drainage volume (ml)	/	190	/	/	/	/	/	/	/
Case 7									
Body temperature	Normal	Normal	Normal	Normal	Normal	Normal	Normal	Normal	Normal
GCS	13	15	15	15	15	15	15	15	15
Pupil diameter (left,right) (mm)	3, 3	3, 3	3, 3	3, 3	3, 3	3, 3	3, 3	3, 3	3, 3
Pupilary light reflex	++	++	++	++	++	++	++	++	++
Postoperative drainage volume(ml)	/	280	/	/	/	/	/	/	/

*GCS* Glasgow Coma Scale. Pupilary light reflex: *−* disappear, *+* slow, *++* sensitive

## Discussion

The rapidly aging population has contributed to an increasing number of elderly patients in China. Once these patients suffer TASDH, a large craniotomy is usually considered as the first-line treatment for TASDH. However, several factors including the old age, APT, and/or comorbid burden need attentions before appropriate surgical procedures were performed. It was reported that TDC could help to prevent subsequent brain injury, assist in stabilizing the clinical condition, and improve the outcome of TBI patients, and it has been chosen for saving valuable time in the patients with TASDH before the evacuation of subdural hematoma via large craniotomy or decompressive craniectomy [1].

It was reported that age ≥ 75 years and TASDH were poor prognostic factors in TBI patients [6]. In our study, there were four patients with age ≥ 75 years, which was the reason that the family members of the patients refused large craniotomy or decompressive craniectomy. Furthermore, the other risk factors, including hypertension, diabetes mellitus, cognitive dysfunction, CI, CHF, and cardiopulmonary complications, are more common in the elderly patients, which would contribute to the poor outcome following TBI.

Because of increased prevalence of the cardiovascular and cerebrovascular disease, there is an increasing number of patients on APT, which has shown a clear benefit in secondary prevention and a possible advantage in primary prevention [7–9]. Once these patients suffer traumatic brain injury (TBI), they will be prone to develop TASDH due to drug-induced impairment of platelet function [10]. It was reported that the pre-injury APT, which was related to the postoperative intracranial hemorrhage, was associated with three times higher mortality among the elderly [11, 12]. Furthermore, two complications such as difficult hemostasis [13] and postoperative intracranial hemorrhage [14] contributed to the poor outcome in the TBI patients. Therefore, it was recommended that all APT should be stopped more than 5 days prior to neurosurgical operation [15]. However, these patients required an urgent decompressive craniectomy, which cannot be delayed to the time when the effect of antiplatelet drugs on hemorrhagic complications disappears. Hence, it still poses a therapeutic challenge for neurosurgeons. Here, we show that the two TASDH cases on APT could be safely and successfully removed by TDC without delay.

The security and effectiveness of TDC, which was a simple, fast, and minimally invasive surgical procedure, have been confirmed in numerous clinical studies [1–3]. It seems that TDC is a widely practiced technique in the treatment of chronic subdural hematoma [16], the surgical operation could be performed at the bedside, and the mean operation time was only 8.9 min [4]. The mean duration of TDC in this study was 14.4 min (6–19 min). Therefore, TDC was more suitable to apply for emergency situations, and when it was combined with other surgical treatment such as subsequent craniotomy, it would provide a more safe, feasible, and effective surgical option than craniotomy only [1]. However, few reports showed that acute subdural hematomas were successfully removed with TDC alone or combined with the instillation of urokinase. Most of us think that blood clot cannot be easily drained out via a closed drainage system. Nevertheless, in our study, mixed high-low densities in the four TASDH patients indicated that there was a non-coagulable blood beneath the dura mater, which could be easily extracted during operation. While high densities in the three patients were not easily extracted, the hematoma aspiration could be performed by the instillation of urokinase for the removal of the residual subdural hematoma, and it could be repeated every 6–8 h at bedside [5]. Fortunately, as shown in the postoperative brain CT scan, all cases including the three cases with high densities revealed the satisfactory clearance of TASDH. Finally, all of patients were discharged uneventfully and have favorable outcomes at 6 months after surgery.

It was reported that injury/surgery time interval was a risk factor in association with preserving the bone flap [17]. In addition, there existed different degrees of brain atrophy in the elderly. Therefore, as long as partial removal of the subdural hematoma was performed rapidly and postoperative intracranial pressure (ICP) was controllable, it is possible to retain the bone flap, and the patient’s life could be saved [18].

Certainly, there were several limitations since this was a retrospective case series study. And we did not perform intracranial pressure monitoring for these patients through this drainage system, which could provide objective evidence for a further large craniotomy or decompressive craniectomy. Lastly, our conclusion were drawn from a retrospective and very small sample size (seven cases); hence, the ultimate safety and efficacy need to be further confirmed in a large sample size.

## Conclusion

To the best of our knowledge, we firstly reported that TASDH in the elderly could be safely and effectively treated by TDC. Several complications such as postoperative epilepsy, external cerebral herniation, intracranial hematoma, and subdural effusion, which occurred after decompressive craniectomy, could be prevented or minimized. There were no cases of acute rebleeding and intracanial infection after TDC. No cases presented with chronic SDH at the ipsilateral side within 6 months after being treated by TDC alone or combined with the instillation of urokinase. Therefore, TDC was a possible alternative for the elderly.

## Abbreviations

APT: Antiplatelet therapy
CHF: Congestive heart failure
CI: Cerebral infarction
CT: Computed tomography
GCS: Glasgow Coma Scale
mRS: Modified Rankin Scale
TASDH: Traumatic acute subdural hematoma
TDC: Twist drill craniostomy

## References

[1] Xiao B, Wu FF, Zhang H, Ma YB (2012). A randomized study of urgent computed tomography-based hematoma puncture and aspiration in the emergency department and subsequent evacuation using craniectomy versus craniectomy only [J]. J Neurosurg.

[2] Barrett RJ, Hussain R, Coplin WM, Berry S, Keyl PM, Hanley DF, Johnson RR, Carhuapoma JR (2005). Frameless stereotactic aspiration and thrombolysis of spontaneous intracerebral hemorrhage [J]. Neurocrit Care.

[3] Vespa P, McArthur D, Miller C, O'Phelan K, Frazee J, Kidwell C, Saver J, Starkman S, Martin N (2005). Frameless stereotactic aspiration and thrombolysis of deep intracerebral hemorrhage is associated with reduction of hemorrhage volume and neurological improvement [J]. Neurocrit Care.

[4] Lu J, Shen D, Hu F, Zhou J, Lan F, Guo D, Liu T (2015). An improved electronic twist-drill craniostomy procedure with post-operative urokinase instillation in treating chronic subdural hematoma [J]. Clin Neurol Neurosurg.

[5] Montes JM, Wong JH, Fayad PB, Awad IA (2000). Stereotactic computed tomographic-guided aspiration and thrombolysis of intracerebral hematoma : protocol and preliminary experience [J]. Stroke..

[6] Prasad GL, Anmol N, Menon GR (2018). Outcome of traumatic brain injury in the elderly population: a tertiary center experience in a developing country [J]. World Neurosurg.

[7] Gouya G, Arrich J, Wolzt M, Huber K, Verheugt FW, Gurbel PA, Pirker-Kees A, Siller-Matula JM (2014). Antiplatelet treatment for prevention of cerebrovascular events in patients with vascular diseases: a systematic review and meta-analysis [J]. Stroke..

[8] Fix ML, Lum MM, Bohan JS (2015). Does Clopidogrel plus aspirin dual therapy reduce risk of stroke in patients at high risk for stroke? [J]. Ann Emerg Med.

[9] Jain N, Hedayati SS, Sarode R, Banerjee S, Reilly RF (2013). Antiplatelet therapy in the management of cardiovascular disease in patients with CKD: what is the evidence? [J]. Clin J Am Soc Nephrol.

[10] Beynon C, Hertle DN, Unterberg AW, Sakowitz OW (2012). Clinical review: traumatic brain injury in patients receiving antiplatelet medication [J]. Crit Care.

[11] Ohm C, Mina A, Howells G, Bair H, Bendick P (2005). Effects of antiplatelet agents on outcomes for elderly patients with traumatic intracranial hemorrhage [J]. J Trauma.

[12] Mak CH, Wong SK, Wong GK, Ng S, Wang KK, Lam PK, Poon WS (2012). Traumatic brain injury in the elderly: is it as bad as we think? [J]. Curr Transl Geriatr Exp Gerontol Rep.

[13] Moringlane RB, Keric N, Freimann FB, Mielke D, Burger R, Duncker D, Rohde V, Eckardstein KLV (2017). Efficacy and safety of durotomy after decompressive hemicraniectomy in traumatic brain injury [J]. Neurosurg Rev.

[14] Flint AC, Manley GT, Gean AD, Hemphill JC, Rosenthal G (2008). Post-operative expansion of hemorrhagic contusions after unilateral decompressive hemicraniectomy in severe traumatic brain injury [J]. J Neurotrauma.

[15] Joo MS, Ahn BM, Kim HJ, Mun HS, Kang MK, Choi SH, Park MJ, Song WK, Lee NH, Cho JR (2014). Evaluation of feasible timing of elective noncardiac procedure after antiplatelet discontinuation in patients treated with antiplatelet agents [J]. J Investig Med.

[16] Chari A, Kolias AG, Santarius T, Bond S, Hutchinson PJ (2014). Twist-drill craniostomy with hollow screws for evacuation of chronic subdural hematoma [J]. J Neurosurg.

[17] Wang X, Zhang R, Tang Z, Liu J, Yang S, Luo W, Wang J, Wei Y, Li J (2014). Factors influencing the decision to retain or remove the bone flap of adult patients with traumatic brain injury: a retrospective study [J]. Turk Neurosurg.

[18] Nguyen HS, Janich K, Sharma A, Patel M, Mueller W (2016). To retain or remove the bone flap during evacuation of acute subdural hematoma: factors associated with perioperative brain edema [J]. World Neurosurg.

